# Automated seamless DNA co-transformation cloning with direct expression vectors applying positive or negative insert selection

**DOI:** 10.1186/1472-6750-10-56

**Published:** 2010-08-09

**Authors:** Natacha Olieric, Melanie Kuchen, Sandro Wagen, Marion Sauter, Stephanie Crone, Sonia Edmondson, Daniel Frey, Christian Ostermeier, Michel O Steinmetz, Rolf Jaussi

**Affiliations:** 1 Paul Scherrer Institut, Biomolecular Research, Villigen PSI, Switzerland; 2 Institute of Biotechnology, Zurich University of Applied Sciences, Wädenswil, Switzerland; 3 Novartis Institutes for Biomedical Research, Basel, Switzerland

## Abstract

**Background:**

Molecular DNA cloning is crucial to many experiments and with the trend to higher throughput of modern approaches automated techniques are urgently required. We have established an automated, fast and flexible low-cost expression cloning approach requiring only vector and insert amplification by PCR and co-transformation of the products.

**Results:**

Our vectors apply positive selection for the insert or negative selection against empty vector molecules and drive strong expression of target proteins in *E.coli *cells. Variable tags are available both in N-terminal or C-terminal position. A newly developed β-lactamase (ΔW290) selection cassette contains a segment inside the β-lactamase open reading frame encoding a stretch of hydrophilic amino acids that result in a T7 promoter when back-translated. This position of the promoter permits positive selection and attenuated expression of fusion proteins with C-terminal tags. We have tested eight vectors by inserting six target sequences of variable length, provenience and function. The target proteins were cloned, expressed and detected using an automated Tecan Freedom Evo II liquid handling work station. Only two colonies had to be picked to score with 85% correct inserts while 80% of those were positive in expression tests.

**Conclusions:**

Our results establish co-transformation and positive/negative selection cloning in conjunction with the provided vectors and selection cassettes as an automatable alternative to commercialized high-throughput cloning systems like Gateway^® ^or ligase-independent cloning (LIC) .

## Background

The use of recombinant DNA technologies is nowadays spread through most laboratories conducting research in life sciences and the applications, including gene expression systems, tend towards being more parallelized. The classical protocols to join DNA fragments by restriction and ligation as well as most techniques employing recombination depend on the presence of specific short sequences at or around the joining regions. However, only completely unrestricted sequence joining will permit us to construct the desired DNAs exactly the way we imagine. The techniques to achieve this are pretty much available today [1,2] and include use of homologous recombination in intact cells [3,4] as well as enzyme mixtures to join the vector and insert DNA *in vitro (commercialized enzyme mixes like In-Fusion, Clontech or ClonEZ, Genscript)*. Mating-assisted genetically integrated cloning (MAGIC, [3]), the perhaps most elegant of the *in vivo *systems, is currently not far enough developed to be broadly applicable and the *in vitro *systems that are distributed by companies are expensive.

The original success with a cloning system employing positive selection [for a review, see [5]] after *in vivo *recombination of inserts in a specific expression vector [4] encouraged us to develop a series of expression vectors relying on a positive or negative selection principle. A positive selection for the insert results whenever the cloning leads to the creation of an additional resistance. The term 'negative selection' is used here to describe cloning systems with vectors that contain the ccdB cell-death gene which is replaced by the cloned insert, *i.e*. the selection eliminates the vector molecules without insert.

Because the trend to parallelization calls for automatable techniques we developed a very robust cloning system that is fully automatable. The initial expression screening of the eight vectors was carried out with enhanced green fluorescent protein and scored positive for all of them (data not shown). In the following, automated cloning and expression screening was conducted with six different target proteins which were known to us to be well expressed. The cloning and expression procedures proved exceptionally robust and all vectors showed high expression comparable to commercially available T7 vectors.

## Methods

### Vector and insert DNA preparation

**Figure 1:**
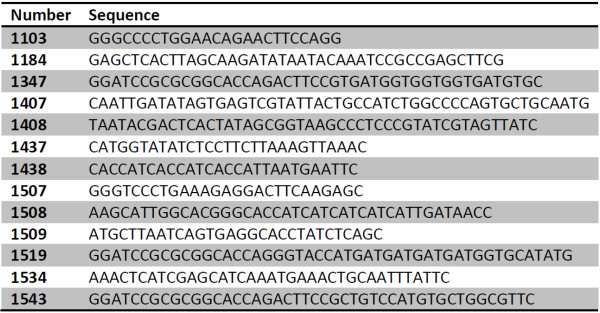
**Sequences of primers for vector amplification**. For use of primers, see figure 2.

**Figure 2:**
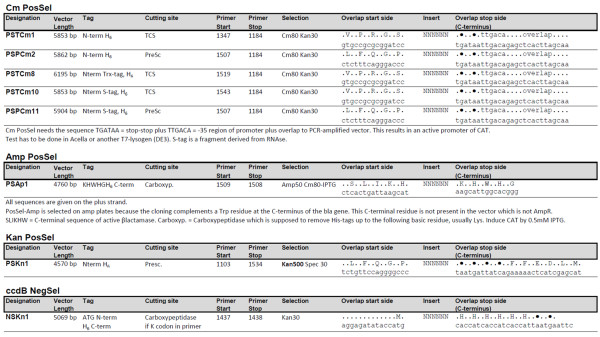
**Features of expression vectors**. All vectors except PSAP1 (backbone is pQE80L, Qiagen, Hilden, Germany) and PSKn1 (backbone is pET28c, Novagen, San Diego, CA, USA) are derived from pET47b (Novagen). Start and Stop primers are used to linearize the vectors for insertion of target sequence by co-transformation. The overlaps with the target PCR are indicated. The sequences of the vector template plasmids are available on request. The nomenclature permits fast recognition of the features of the vectors: The names start with PS for positive selection with chloramphenicol (Cm80 = Chloramphenicol 80 mg/L), ampicillin (Amp) or kanamycin (Kan) and NS, for negative selection with ccdB, respectively. The proteolytic cleavage site is indicated by T for thrombin and P for prescission proteases. The tag(s) are indicated and finally the antibiotic resistance is given followed by a number. The concentration of the antibiotics can vary greatly depending on the vector's copy number per cell.

All PCR primers were synthesized by Microsynth (Balgach, Switzerland) and used without further purification. Oligonucleotide sequences and uses are given in Figures 1 and 2, respectively. Template DNA for PCR was prepared by extraction from transformed Mach1 cells or ccdB survival cells (Invitrogen, Rotkreuz, Switzerland) using the GeneJet DNA minipreparation kit (Fermentas, Vilnius, Lithuania). About 2 ng of DNA were used as template for PCR. We used Phusion^® ^polymerase (Finnzymes Oy, Espoo, Finland) as described [6]. Most proofreading DNA polymerases may be used, however, it is vital to avoid polymerases with terminal transferase activity like Taq that lead to A-tailing of the 3' ends of the PCR product. Before co-transformation both vector and insert were linearized by PCR. The excess of primers was removed by reaction cleanup on Minelute columns (Qiagen, Hilden, Germany). For vector preparations requiring more DNA, several PCR reactions were pooled and purified on larger spin columns (25 μg capacity, Fermentas, Vilnius, Lithuania). In general, cloning success depends on quality of the PCR product which should show a single band on agarose gel after electrophoresis. Gel purification of the PCR product and digestion of the templates with DpnI are not necessary (unless a template plasmid carries both resistances).

### Co-transformation cloning using *E.coli *Mach1 cells

All plasmids were constructed by *in vivo *joining of PCR products with overlapping ends (about 15 bp) by a technique which we call co-transformation cloning. The *E.coli *strain Mach1 yields most colonies, but a few other strains like DH5alpha and Top10 work also. Co-transformation employs chemically competent cells [7] yielding 10^7 ^or more colonies per μg plasmid. Per co-transformation 200 ng of vector plus 50-500 ng of insert were mixed and the competent cells added to the DNA mixture which was less than 10% of the cell volume (50 μL cells). The protocol is standard: 30 minutes on ice, 45 s at 42°C, 1 min on ice and then addition of 4 volumes SOC medium [8]. In contrast to other protocols, a longer recovery time of 2 h was necessary to complete the end joining reaction before antibiotic selection was applied (Figure 2). Co-transformation works well with inserts up to 1.5 kb length. For longer inserts or cloning without positive or negative selection we use the ClonEZ kit (Genscript, Piscataway, NJ, USA).

### Automation and general molecular biology techniques

All experimental procedures were carried out using a TECAN Freedom Evo II liquid handling workstation. The only procedures performed by hand were colony picking and insert DNA purification with the Qiagen Minelute reaction cleanup kit which are, however, both automatable (Reference [9], Qiacube from Qiagen).

The basic techniques applied for construction of the vectors (not described in detail) are from the Molecular Cloning Handbook [8]. DNA fragments were analyzed by fast agarose gel electrophoresis [10,11]. DNA restriction or modification enzymes were from Fermentas (Vilnius, Lithuania) or New England Biolabs (Ipswich, MA, USA). For colony-PCR the Go-Taq Mastermix from Promega (Madison, WI, USA) was used. Cells were spread on agar plates by shaking on the TECAN workstation. Two colonies per target were picked and inoculated into 3 mL of 2xTY media with antibiotics. The cells were grown overnight at 37°C and spun down at 1900 rpm for 10 min. The plasmids were prepared on our TECAN workstation using the NucleoSpin Robot 96 Plasmid Kit. The plasmid was eluted with 200 μL of elution buffer and the yield was quantified by UV absorption.

### Protein expression

All methods were carried out based on standard protocols [8] and are briefly described: The expression plasmids were transformed into *E. coli *expression strains (BL21(DE3) for EB1, Clip170-CapGly, TTL, CLIP170-full, PKNG and Acella for AAV2-VP3, respectively) and selected on LB-agar plates with antibiotics. Pre-cultures were grown over night at 30°C in deep 24-well blocks inoculating 4 mL LB. Expression cultures were started the next day by adding 200 μL pre-culture to 4 mL LB media. The cultures were grown at 37°C until the OD_600 _reached 0.4 and then moved to a 20°C incubator. The expression was induced 30 min later with 1 mM IPTG for Clip170-CapGly, EB1, CLIP170, VP3 and TTL or 0.1 mM IPTG for PKNG. The cells were harvested after overnight growth at 20°C.

The 4 mL cultures were pelleted and resuspended into 1 mL lysis buffer (50 mM HEPES pH8, 500 mM NaCl, 10% glycerol, 10 mM imidazole). The cells were lysed by sonication. The cell extracts were centrifuged for 10 min at 15,000 × g at 4°C. The soluble fractions were loaded onto 400 μL NiNTA IMAC resin (Ni SepharoseTM High Performance, GE Healthcare) in a 96 well filter plate (Novagen) pre-equilibrated with HEPES pH8, 500 mM NaCl, 10% glycerol, 10 mM imidazole. The beads were washed 3 times with 1 mL of the above buffer and the proteins eluted with 200 μL 50 mM HEPES pH8, 500 mM NaCl, 10% glycerol, 500 mM imidazole. The purified proteins were analysed by SDS-PAGE and Western blotting with anti-penta His antibodies (Qiagen).

## Results

### Basic strategies and development of cloning vectors

**Figure 3:**
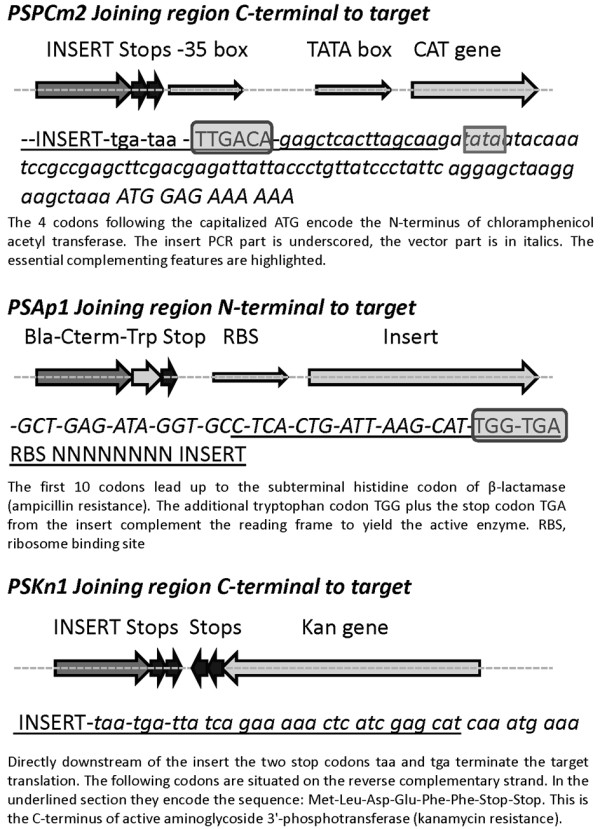
**Schemes of applied insert selections**. For each selection type one vector example is shown. Positive selection with chloramphenicol: Vector with inactive promoter, upon insertion of target the -35 box is introduced and the promoter is active. Positive selection with kanamycin: By insertion of the target PCR a new C-terminal Phe residue is introduced which activates the kanamycin kinase. Wild Type kanamycin kinase has two Phe residues at the C-terminus. Positive selection with ampicillin: Similar to the kanamycin selection, the missing C-terminal Trp residue of beta-lactamase is complemented by the insert cloning. Negative selection with ccdB: The cassette with the cell killer gene ccdB is replaced by the desired insert.

**Figure 4:**
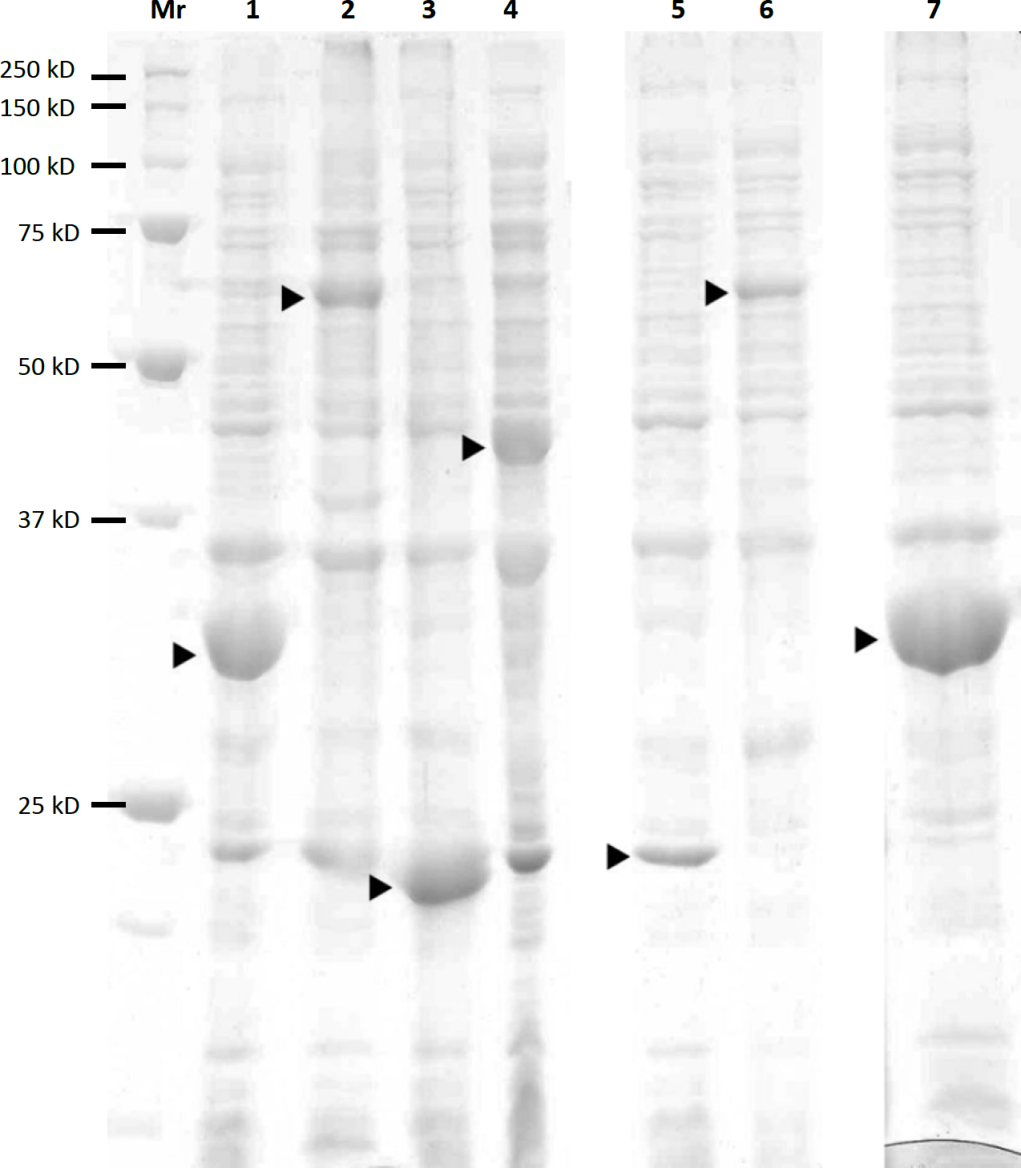
**High expression levels are reached with most of the vectors**. The cell pellets from expression cultures were extracted with SDS-gel sample buffer and run on 12% SDS_PAGE. From left to right: Mr markers; 1, PSTCm1-EB1; 2, PSPCm2-VP3; 3, PSTCm8-CapGly; 4, PSTCm8-TTL; 5, PSTCm11-CapGly; 6, PSKn1-Clip170; 7, NSkn1-EB1. The desired expression product is represented by the strongest band in the corresponding lane with the total cell extract. Only the vector PSAp1 gave less vigorous expression (not shown).

Three strategies are used to achieve positive selection for the target insert (Figure 3) by creating a new antibiotic resistance which is coupled to the correct orientation and terminal sequence of the insert DNA. A fourth strategy is applied to get negative selection against the vector backbone which contains the ccdB cell death gene that is removed during the cloning procedure. The vectors permit a high level of target expression in *E.coli *(Figure 4) and are mostly derivatives of pET47b (PSTCm1, PSPCm2, PSTCm8, PSTCm10, PSPCm11, NSKn1). Several of the vectors come in two versions, one with an N-terminal thrombin- and the other with a prescission-protease cleavage site downstream of the his_6_-tag. This is achieved by amplifying the same template plasmid with different primer sets. In one of the vectors, the his_6_- tag is positioned C-terminally of the target protein. The additional thioredoxin- and RNAse S-tags are intended to increase the yield of expression [12].

The construction of new vectors relies on the insertion of a selection cassette as amplified from one of the expression constructs. We can complement three different defective antibiotic resistance genes for insert selection with chloramphenicol, kanamycin or ampicillin (Reference [4] and Figure 3, see also 'Additional file 1' for nucleotide sequences of the vectors). For example, the chloramphenicol selection-cassette was amplified such as to contain the CAT gene plus its active promoter including the -30 TTGACA sequence [13] and inserted into the vector backbone. The desired tag was also inserted by PCR-mutagenesis using two primers to amplify the whole plasmid along with the tag sequence. The primers are designed to create 15 bp of identical sequence in the region of the tag at each end of the linear PCR product. The PCR product is digested with Dpn I and transformed into *E.coli *Mach1 cells. These cells can circularize the plasmid by recombination of the short terminal repeats at the ends of the PCR product. This kind of mutagenesis is efficient; more than 90% of the clones are correct. If two DNA fragments have to be joined, corresponding overlaps are designed at the ends of the fragments which are co-transformed into Mach1 cells.

The vector PSAp1 is derived from pQE80L (Qiagen) and permits positive selection with ampicillin for a target with C-terminal tag fusion. In order to activate the silent chloramphenicol acetyl transferase gene in pQE80L, the lambda terminator downstream of the T5 promoter was deleted. This version of pQE80L became resistant to chloramphenicol in presence of 0.5 mM IPTG. The plasmid was amplified with two primers (#1407 and #1408) containing a T7 promoter sequence in the 5' attachments to the annealing region. The insertion occurs into the second C-terminal loop of beta-lactamase (see 3D-structure NCBI structure database, molecule 1BTL). The reading frame in the T7 promoter sequence region was chosen such as to encode hydrophilic amino acids. The PCR product was digested with Dpn I and transformed into *Mach1 *cells. After verifying this mutagenesis by DNA sequencing, a positive clone was tested for ampicillin resistance, which proved to be similar to the wild type plasmid. Several other constructs with promoter insertions using different borders or insertions in the first C-terminal loop or split gene approaches led to inactivation of beta-lactamase. The plasmid with ampicillin resistance was amplified with two primers (#1470 and #1471) in order to introduce a C-terminal His_6_- tag and to delete the C-terminal tryptophan residue of β-lactamase (called ΔW290). This plasmid is designated #790, is ampicillin-sensitive and used as a template to amplify the linear positive selection vector PSAp1. As opposed to the situation shown in Ref. [4] the β-lactamase gene is located upstream of the insert and has the same orientation as the insert. This is possible because of the newly inserted T7 promoter which was engineered into the β-lactamase gene and which now drives the expression of the target. Because the T7 promoter lies within a transcribed gene, it is less vigorously active than a T7 promoter in a non-transcribed region of a standard T7 expression vector. Although we have observed about 10 fold lower expression levels with this vector compared to standard T7 vectors, it may come in handy if attenuated target expression is desired, e.g. for expression of membrane proteins.

Negative selection in the vector NSKn1 is due to a ccdB cell death gene in the vector which has to be grown in ccdB survival cells. The sequence of the insertion including the ccdB gene is given in the 'Additional file 1'. This toxic gene is replaced by the insert protein; the construct grows now in Mach1 cells. A primer pair is used to amplify the linear vector excluding the ccdB gene (Figures 1 and 2, primers 1437 and 1438). This vector backbone is then co-transformed with the insert DNA which overlaps with the vector ends at the termini of the PCR fragment. The pET47b-derived vector was linearized by PCR (primers #1410 and #1411). The ccdB cassette with the appropriate ends was optimized for expression in *E.coli *using the Gene Designer program (DNA 2.0 Inc.) and synthesized by Genscript. This DNA (790 bp sequence, see 'Additional file 1') was PCR-amplified (primers #1412 and #1413) and joined with the vector by treating with the ClonEZ kit (Genscript) and transformed into ccdB survival cells because the ccdB survival cells were not able to recombine the PCR products upon co-transformation.

The only vector employing positive selection by kanamycin has a backbone from the plasmid pRSF (Merck Biosciences), is streptomycin resistant and can be transformed and maintained in an *E.coli *strain which simultaneously harbors a second plasmid with a colE1 origin like the pET-derivatives using chloramphenicol or the pQE80 derivative using ampicillin for selection. Therefore two proteins can be co-expressed in the same host cell.

### Construction of expression plasmids by co-transformation

**Figure 5:**
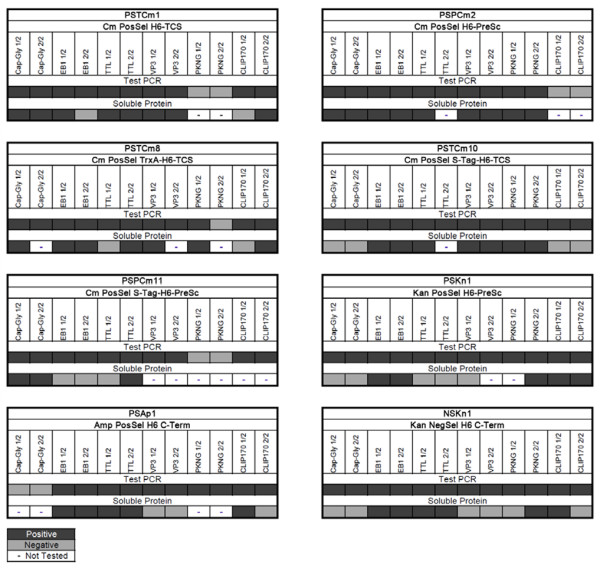
**Overview of cloning and expression results**. All cloning results with the described vectors and targets are summarized. Two colonies were picked for each vector-target combination and analyzed in order to apply a stringent test to the cloning and expression systems. Top lanes state vector name and design features; vertical lanes indicate target proteins; shaded lanes indicate experimental results; code see below; upper lane with cloning result, ie. PCR with insert primers; lower lane with expression result, ie. soluble protein after mini-IMAC target purification. Positive Test PCR means a clearly visible band of the expected size on agarose gel. Positive for soluble protein means a clearly visible band on the Coomassie-stained SDS-PAGE at the correct size in the fraction after purification by Ni-NTA column chromatography. Designations: 1/2 and 2/2 indicate first and second clone that were picked; CapGly, Mr = 10 KDa, CAP-Gly domain 1 of human CLIP170, accession number NP_002947; EB1, full length of human EB1 protein, Mr = 32 KDa, accession number AAC09471; TTL, Mr = 48 KDa, full length human tubulin-tyrosin ligase, accession number NP_714923; VP3, Mr = 62 KDa, full length of adeno-associated virus capsid protein 3, accession number AF043303, synthetic sequence, see 'Additional file 1'; PKNG, 78 KDa, full length of the *M. tuberculosis *serine/threonine-protein kinase G, accession number NP_214924; CLIP170, Mr = 54 KDa, fragment of human CLIP170 fused with a GCN4 sequence, accession number NP_002947.

All vectors and inserts were linearized by PCR on a TECAN workstation using 96-well microtiter plates. The PCR products display a set of standardized cloning overhangs, usually a sequence encoding the proteolytic cleavage site and another one in the positive selection region of the vector. Some of the eight vectors share the cloning overhang pairs (Figure 1), *i.e*. each target had to be amplified with five different primer pairs to permit cloning of all vector-insert permutations (constructs summarized in Figure 2). The pipetting of vector-insert pairs, co-transformations and plating onto 12-well agar plates were again performed by the TECAN workstation. After overnight incubation at 37°C we observed 10-50 colonies per well. In rare cases without colonies the leftover cells could be spread on 10 cm diameter agar dishes and then gave a few up to 50 colonies. Over 90% of the cloning assays resulted in enough colonies to go on. To stringently test the cloning and expression efficiency, only two colonies per target-vector combination were picked and grown in 2xTY broth with antibiotics in 24-well plates. The plasmid preparation was performed the next morning on the TECAN workstation. The resulting DNAs (70-150 ng μL^-1^) were used as template for diagnostic PCRs with the same primers as those for insert amplification. Usually both or at least one of the clones scored positively in 85% of the cases (Figure 5). A series of the positive clones was subjected to restriction mapping which indicated that all clones were correct. Eight clones were randomly selected and subjected to DNA sequencing. All of them contained the expected insert in the proper orientation in the chosen vector.

During the last two years many cloning experiments by co-transformation without positive selection have been performed in our laboratory. In these cases a success rate of 10-90% was achieved. Thus, these methods are also suitable for everyday seamless cloning without applying any selection for the correct clones. The main limitation seems to be the vector and insert length. The larger the DNAs, the less frequently they will be co-transformed. Treatment of the PCR products with the ClonEZ kit increases both the number of colonies and the rate of success. The SLIC method [14] can be used as a backup procedure. We suggest using a variant (see 'Additional file 1') of the published protocol which is unreliable in our hands. We feel that in conjunction with these two rescue methods most standard cloning applications are covered by the protocols described here. This means easy seamless cloning with free choice of the cloning overhangs is now possible at low cost for the majority of basic cloning experiments. All methods described in this publication are fully automatable for high-throughput applications. With well-expressible targets of less than 1.5 kb length it is sufficient to pick randomly two colonies to reach a 70% success rate in expression of the target. If a higher success rate is required, more colonies can be picked. This may be necessary when cloning target DNA by direct PCR amplification from libraries [6]. A recently published method to deplete shorter PCR products in mixtures [15] may be helpful in these cases.

### Expression yields high level of soluble protein

**Figure 6:**
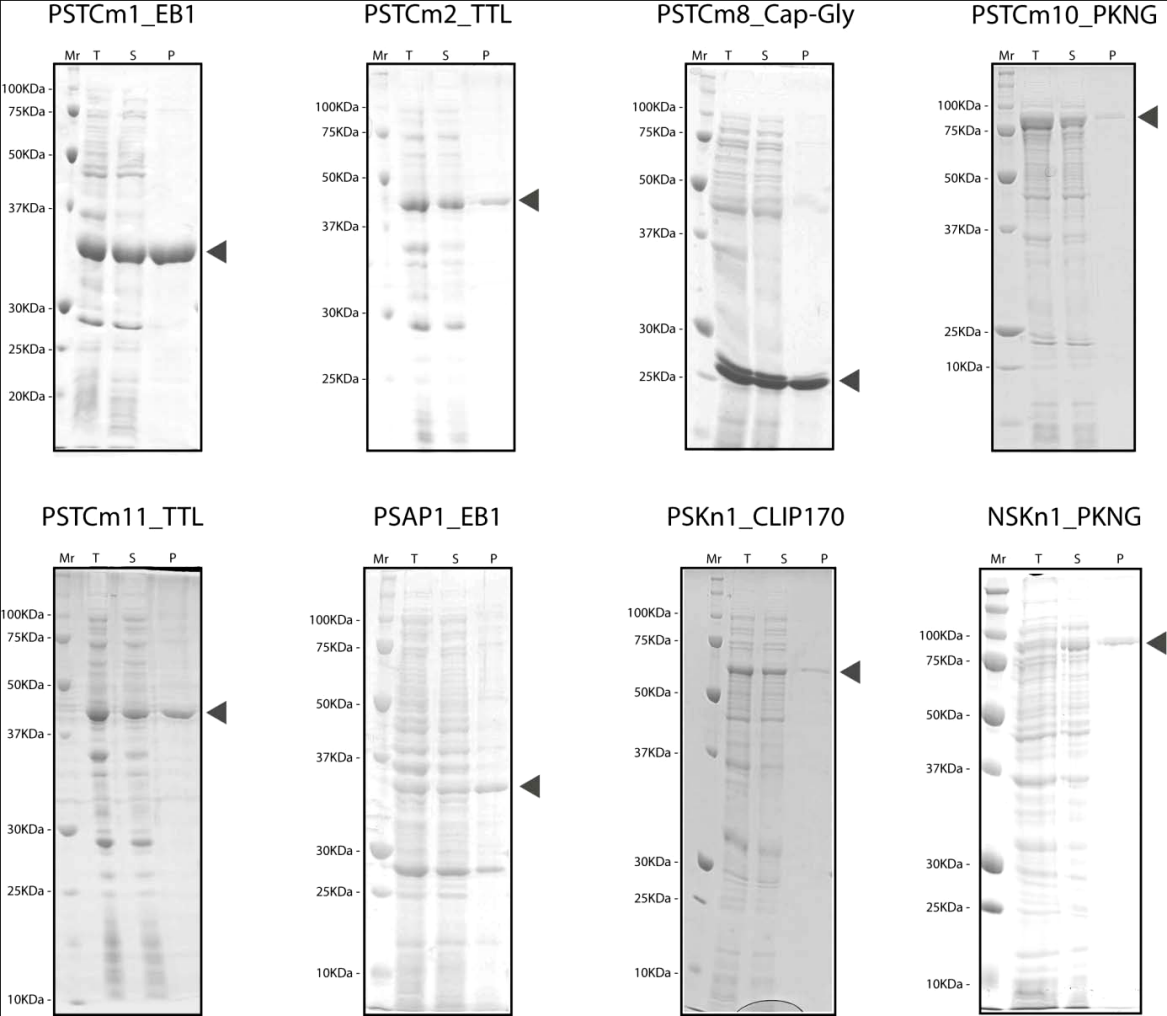
**Small scale purification of expression products**. The cells of 4 ml culture were pelleted and disrupted by sonification. After centrifugation the target proteins in the supernatant were purified by automated batch IMAC and run on 12% SDS-PAGE. In each panel, total protein T, protein in the supernatant S and purified protein P are given. Typical examples of purifications are shown.

The level of protein expression attained in *E.coli *cells by the new vectors (all vectors, except PSAP1) was comparable to that observed with typical commercial expression vectors like pET15, pET28 or pET47 (Figure 4). 80% of the constructs with insert (Figure 5, Test PCR) yielded soluble expression of the target protein as demonstrated by purification of the products by IMAC (Figure 5 and Figure 6, Soluble Protein). Based on sequencing of a few expression-negative examples, we assume that mutations in the regions of the incorporated synthetic primers account for a large proportion of the negative results.

## Discussion

### A facile yet reliable way of molecular cloning

Recently, a library with almost 5,000 cDNAs was subcloned into an expression vector by an elaborate seamless enzyme-free cloning method [16] using intracellular DNA recombination. The inserts were PCR-amplified, gel-purified and mixed with vector that had been linearized by restriction and gel purification. The transformation of the purified vector plus PCR product into very highly competent *E.coli *cells yielded a similar cloning success rate as achieved in this work. In a later version, a positive selection was applied that depends on the use of a specifically engineered cell [3] or a cell with an intact lacZ reporter gene. Due to the gel purification step, this method is currently not amenable to automation. The publication also lacks the demonstration of target expression. Despite these shortcomings, the results confirm the usefulness of the approach as described here.

Automated applications call for robust processes with a cloning success rate exceeding 80% while co-transformation cloning usually yields more than 20% correct clones. Thus, we needed to increase the percentage of correct clones. To achieve this goal all our direct expression vectors for *E.coli *employ positive or negative selection. Judged from our experience with a set of six widely different, but well expressible target genes we conclude that our vectors perform as well as commercial standard expression vectors. This is no surprise because the vectors have been built based on well-established vector backbones. The new element is the positive selection cassette which was introduced by the co-transformation technique. PCR-amplified activated versions of the cassettes can be used for vector construction and later reversion to a selection-negative vector by site-directed mutagenesis. In this way, most plasmids can be quickly converted to positive selection vectors. The level of residual resistance to the selective antibiotic depends on the copy number of the used plasmids. Hence, we suggest to titrate the resistance of the vector and to compare it to a construct which contains a selectable insert.

## Conclusions

The increasing demand for genetically engineered proteins prompted others [17] and us to develop a robust, simple, low-cost approach for rapid target expression cloning on automated platforms. Most published cloning systems require *in vitro *modification of the insert DNA and the vector DNA with techniques beyond a simple PCR. The here introduced methods lead to efficient assembly of direct expression plasmids starting with purified PCR-products both for the vector and the insert DNA. The vector and insert PCR products have 13-20 bp long short regions of identity at their respective ends. No further *in vitro *steps are required to construct the expression clones. The recombination of the matching ends occurs inside the transformed *E.coli *cells. We have called this process "co-transformation cloning". Our results establish co-transformation and positive selection cloning in conjunction with the provided vectors and selection cassettes as an alternative to high-throughput cloning systems like Gateway or ligase-independent cloning (LIC).

## Authors' contributions

NO carried out most protein expressions and wrote part of the publication. MK, SW, MS, SC, and SE established the necessary genetic constructs and helped with their design. DF was responsible for the automation and was involved with all experimental steps. CO and MOS contributed original ideas and gave support on techniques as well as writing. JR contributed with ideas, coordinated the work and wrote most of the manuscript. He was also engaged in development of the original assays by hand. All authors read and approved the final manuscript.

## Supplementary Material

Additional file 1 **Supplementary materials**. Click here for file
